# In-Situ Fabrication of Electroactive Cu^2+^-Trithiocyanate Complex and Its Application for Label-Free Electrochemical Aptasensing of Thrombin

**DOI:** 10.3390/bios13050532

**Published:** 2023-05-10

**Authors:** Zehao Wang, Ningning Gao, Zhenmao Chen, Feng Gao, Qingxiang Wang

**Affiliations:** 1 College of Chemistry, Chemical Engineering and Environment Science, Fujian Provincial Key Laboratory of Modern Analytical Science and Separation Technology, Minnan Normal University, Zhangzhou 363000, China; wzh25739@163.com (Z.W.); gnn6042@163.com (N.G.); czhenmao@163.com (Z.C.); gf1377@mnnu.edu.cn (F.G.); 2 Anhui Laboratory of Molecule-Based Materials, Anhui Normal University, Wuhu 241000, China

**Keywords:** trithiocynate, Cu^2+^, electrochemical, aptasensor, thrombin

## Abstract

The preparation of an electroactive matrix for the immobilization of the bioprobe shows great promise to construct the label-free biosensors. Herein, the electroactive metal-organic coordination polymer has been in-situ prepared by pre-assembly of a layer of trithiocynate (TCY) on a gold electrode (AuE) through Au-S bond, followed by repetitive soaking in Cu(NO_3_)_2_ solution and TCY solutions. Then the gold nanoparticles (AuNPs) and the thiolated thrombin aptamers were successively assembled on the electrode surface, and thus the electrochemical electroactive aptasensing layer for thrombin was achieved. The preparation process of the biosensor was characterized by an atomic force microscope (AFM), attenuated total reflection-Fourier transform infrared (ATR-FTIR), and electrochemical methods. Electrochemical sensing assays showed that the formation of the aptamer-thrombin complex changed the microenvironment and the electro-conductivity of the electrode interface, causing the electrochemical signal suppression of the TCY-Cu^2+^ polymer. Additionally, the target thrombin can be label-free analyzed. Under optimal conditions, the aptasensor can detect thrombin in the concentration range from 1.0 fM to 1.0 μM, with a detection limit of 0.26 fM. The spiked recovery assay showed that the recovery of the thrombin in human serum samples was 97.2–103%, showing that the biosensor is feasible for biomolecule analysis in a complex sample.

## 1. Introduction

Electrochemical biosensors, as one of the earliest kinds of biosensors, have been widely used in the fields of health care, the food industry, agriculture, and environmental monitoring because of their high sensitivity, easy miniaturization, and ability to detect samples in complex systems [1,2,3]. In recent years, aptamers have attracted great attention as the recognition elements in the design and construction of biosensors [4,5]. Compared with the traditional recognition element of antibodies and enzymes, the nucleic acid aptamer (NAA) has comparable or better properties for the construction of biosensors, namely, the aptasensors [6,7]. This can be attributed to the unique versatile features of the NAA. For example, the NAAs can be chemically synthesized with low cost and high precision, and can be modified by various functional groups carrying electroactivity, photoactivity, or biological/chemical affinity [8,9,10,11]. In addition, the NAAs have a wide range of recognition targets from ions and small molecules to macromolecules, and even the whole cells, and in theory, any target can be screened by specific aptamers through the systematic evolution of exponentially concentrated ligands (SELEX) [12,13,14]. What is more, the binding between NAAs and the target is commonly reversible, which enables the aptasensor to be reused through appropriate methods such as heating or elution with buffer [15].

For an electrochemical aptasensor, the conversion of the bio-recognition event into readable electrochemical signals is one of the critical issues. To date, two types of signal output manners have been developed. One is based on the covalent modification of signal tags (such as ferrocene or methylene blue) at the end of the NNAs [16,17,18]. Once binding to the target, the conformation of the NAA is changed, resulting in the variation of the distance between signal tags and the basic electrode. Consequently, the electrochemical signal of the aptasensor is changed. However, this sensing method has the following shortcomings: (1) the process of signal molecular labeling is complex, tedious, and expensive; (2) the NNAs are commonly labeled by a single site at the end, which makes the signal output intensity weak, leading to the poor sensitivity of the aptasensors. Another way of the signal output of electrochemical aptasensor is based on external signal molecules, typically [Fe(CN)_6_]^3−/4−^ [12,19]. When the target binds to the NAA via bioaffinity, a non-conductive complex film is formed on the electrode surface, which hinders the diffusion of [Fe(CN)_6_]^3−/4−^ from the bulk solution to the electrode surface. Thus, the target can be detected by impedance and voltammetry. The process of this method is simple, but it has a high background response. At the same time, the non-specific adsorption of interfering substances on the electrode surface can also lead to impedance or voltammetric response, which is easy to cause false-positive test results.

In recent years, the electrochemical label-free biosensing strategy based on the electroactive interface has attracted increasing attention [12,20,21]. For example, Gao et al. [21] developed a platinum nanoparticle (Pt NPs) decorated carbon nanocages as a signal label to construct a sandwich-type electrochemical aptasensor for thrombin detection. Pt NPs act as a hydrogen peroxide mimetic enzyme to catalyze the reduction of H_2_O_2_, resulting in a signal amplification of the differential pulse voltammetry (DPV) peak current. Indirect detection of different concentrations of thrombin. Our group has also proposed a sensitive electrochemical biosensing strategy based on the hybridization-induced ion-barrier effect on the electroactive sensing interface [22]. In the work, a bifunctional electrode with the nanocomposite of electroactive Prussian blue (PB) and gold nanoparticles (AuNPs) was prepared through a two-step electrodeposition process. Upon specific hybridization of probe DNA with the target, the formed complex induced the ion-barrier effect, which blocked the diffusion of the K+ from the bulk solution to the electrode surface, and accordingly, the voltammetric signal of the PB on the electrode was suppressed. In these electroactive interface-based label-free protocols, the integrated design of the immobilization platform of the biological probe and the signal source not only avoids the labeling of the signal molecules but also greatly expands the signal output intensity as well as the analytical sensitivity because the signal molecules contact with the basic electrode directly.

**Scheme 1:**
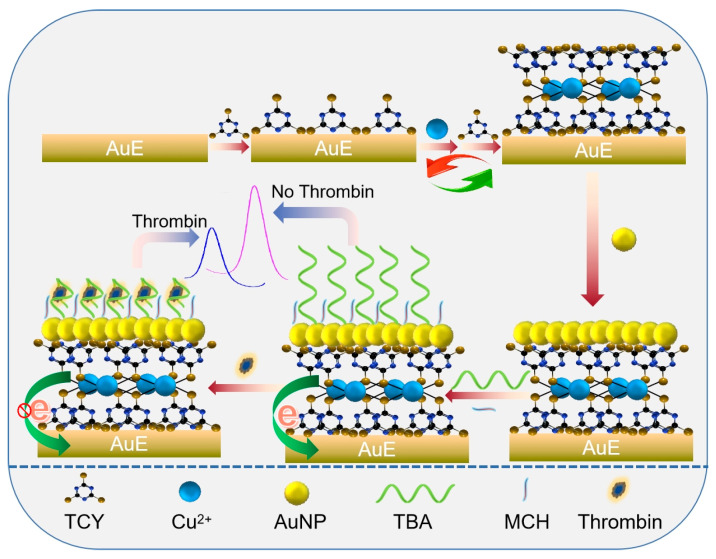
Schematic illustration for the preparation of AuNPs/Cu^2+^-TCY polymer-based aptasensing film on a gold electrode and its analytical application.

Herein, an electroactive metal-organic polymer-based aptasensing platform was fabricated by the repetitive assembly of trithiocyanate (TCY) and copper ion (Cu^2+^) on a gold electrode (AuE), and then the thrombin was applied as the target model to probe the analytical performance of the aptasensor. Scheme 1 shows the fabrication process and working principle of the aptasensor. The TCY was grafted on the gold electrode (AuE) via the Au-S bond, and then the Cu^2+^ and TCY were repeatedly assembled through the coordination chemistry between -SH and Cu^2+^. The Cu^2+^ endows the sensing interface with an excellent electrochemical signal by its redox couple of Cu^2+^/Cu^+^, and the TCY also provides the supporting site for the assembly of the gold nanoparticles (AuNPs) that are used for the immobilization of the thiolated thrombin-binding aptamer (TBA). Upon binding of TBA with the target thrombin, the non-conductive complex was formed on the electrode surface, causing a decrease in the electrochemical response of Cu^2+^-TCY polymer. Additionally, the thrombin was facilely monitored. This work provides a new idea to develop an electrochemical aptasensing strategy for the label-free analysis of thrombin.

## 2. Materials and Methods

### 2.1. Reagents and Apparatus

Tris-(2-carboxyethyl)-phosphine hydrochloride (TCEP), 6-Mercaptohexan-1-ol (MCH) were obtained from Sigma-Aldrich Co., Ltd. (Shanghai, China). Copper nitrate trihydrate(Cu(NO_3_)_2_∙3H_2_O), potassium ferricyanide (K_3_Fe(CN)_6_), potassiunferrocyanide (K_4_Fe(CN)_6_), and the other metal salts were provided by Xilong Chemical Company (Shantou, China). The 25 mM phosphate-buffered saline (PBS, pH 7.0) was provided by Shanghai KangYi Instruments Co., Ltd. (Shanghai, China). Chloroauric Acid (HAuCl_4_·4H_2_O) was achieved from TCI Development Co., Ltd. (Shanghai, China), and the AuNPs stabilized with citrate were prepared according to the procedure reported previously [23]. Albumin from bovine serum (BSA, 66.4 kD), Tris(hydroxymethyl)methyl aminomethane THAM (Tris) was obtained from Sinopharm Chemical Reagent Co., Ltd. (Shanghai, China). Hemoglobin (Hb, 64.5 kD) and the 5′- thiolated thrombin aptamers (TBA) with the sequence of 5′-SH-(CH2)6-GGT TGG TGT GGT TGG-3′ were purchased from Shanghai Sangon Bioengineering Co., Ltd. (Shanghai, China). Thrombin (37.0 kD) was bought from Shanghai Yuanye Biotechnology Co., Ltd. (Shanghai, China). The molar concentrations of the proteins and enzymes are calculated from their molecular weights. All the other chemicals were of analytic reagent grade and obtained commercially. Doubly distilled water (DDW) was used throughout the experiments.

Attenuated total reflection Fourier transform infrared (ATR-FTIR) spectroscopy was performed on a Nicolet iS 10 spectrometer (Waltham, MA, USA). Atomic force microscopy (AFM) measurements were carried out on Bruker Multimode 8 with a NanoScope V Controller (Billerica, MA, USA). Electrochemical measurements were carried out on CHI 650C electrochemical analyzer (Shanghai, China) in connection with a conventional three-electrode system, which includes an AuE (diameter = 3 mm) working electrode modified with different materials, a reference electrode of Ag/AgCl, and a counter electrode of Pt wire.

### 2.2. Fabrication of the Electroactive Cu^2+^-TCY Polymer on AuE

Prior to fabrication, the bare AuE was cleaned through physical polishing, chemical oxidation, and electrochemical oxidation, as reported in our previous method [3]. Following that the cleaned AuE was incubated in 200 μL of 0.01 M TCY for 1 h to assemble the TCY through the Au-S bond. After that, the electrode was washed with DDW to remove the physically absorbed TCY, and thus the TCY-modified electrode (TCY/AuE) was obtained. Thereafter, the electrode was successively treated with 200 μL of 10 mM Cu^2+^ for 1 h and 200 μL of 10 mM TCY for 1 h for three cycles. After each incubation, the electrode was rinsed with DDW, and finally, the electroactive Cu^2+^-TCY polymer-modified electrode, termed p(Cu^2+^-TCY)/AuE, was achieved.

### 2.3. Assembly of AuNPs and Immobilization of the Aptamer

The assembly of AuNPs on the electrode was carried out by incubating p(Cu^2+^-TCY)/AuE into an AuNPs dispersion for 4 h under room temperature with gentle shaking. After being washed by DDW to remove the loosely absorbed AuNPs, the AuNPs modified electrode (AuNPs/p(Cu^2+^-TCY)/AuE) was achieved and was further treated with 200 μL of 10 μM thiolated TBA for 30 min under 37 °C, through which the TBA was immobilized on the electrode by interaction with the AuNPs. Then, the TBA-modified electrode was subjected to 200 μL of 1 mM MCH for 2 h under room temperature to passivate the residual site of AuNPs, from which the non-specific absorption of pollutants on the electrode surface can be prevented [24]. Through the above process, the aptasensor with the electroactive layer of p(Cu^2+^-TCY) was fabricated.

### 2.4. Thrombin Binding and Electrochemical Measurements

The recognition of the aptasensor with the target thrombin was performed by incubation of the aptasensor in thrombin solution with desired concentration for 40 min under ambient temperature. After washing with PBS, the electrode was electrochemically scanned in 25 mM PBS by differential pulse voltammetry (DPV) for sensing analysis with the potential range from −0.1 to 0.5 V.

The electrochemical characterization on the fabrication of the aptasensor was carried out through cyclic voltammetry (CV) and electrochemical impendence spectra (EIS) in the mixture solution of 1.0 mM [Fe(CN)_6_]^3−/4−^ (1:1) and 0.1 M KCl. The potential window in CV was between −0.2 and +0.6 V, and the frequency range in EIS was from 1 to 10^5^ Hz at an applied potential of 0.210 V.

To investigate the practicability of the aptasensor, recovery experiments were conducted in healthy human serum samples. Prior to the detection, the human serum was diluted 2-time with PBS, and then different concentrations of thrombin standard solution were injected. After incubating in the serum samples with the injected thrombin for 40 min, the aptasensor was washed with PBS and then tested in 25 mM PBS with DPV.

## 3. Results and Discussion

### 3.1. Physical Characterization on the Fabrication of Aptasensor

**Figure 1:**
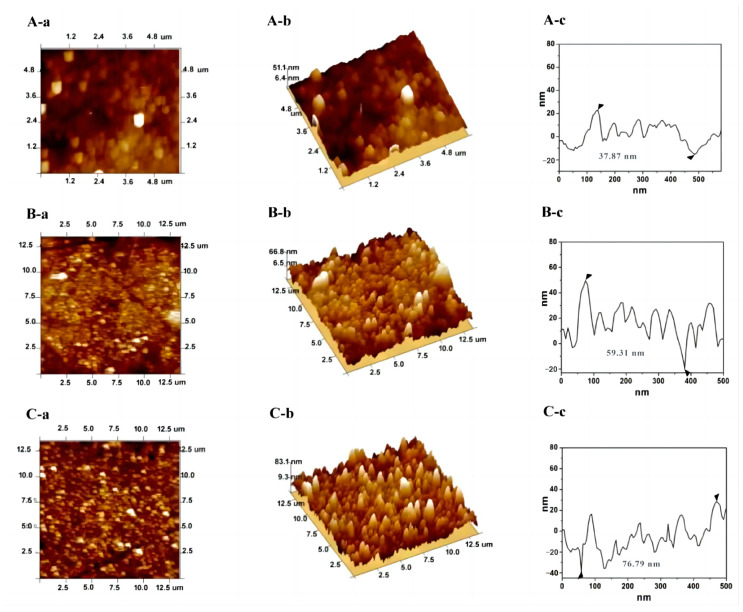
Topographic (**a**), three-dimensional (**b**) and cross-sectional (**c**) AFM images of AuE upon stepwise assembly of p(Cu^2+^-TCY) (**A**), AuNPs (**B**), and MCH/TBA (**C**). The triangle symbols in the c represents the lowest and the highest points in the cross-sectional line.

Atomic force microscopy (AFM) is a high-resolution scanning probe microscopy technology that is applicable for probing the topographic changes of an interface [25,26]. In this work, the fabrication of aptasensor by a successive assembly of p(Cu^2+^-TCY), AuNPs, and MCH/TBA was characterized by AFM. Figure 1 shows the topographic (a), three-dimensional (b), and cross-sectional (c) AFM images of AuE upon stepwise assembly with p(Cu^2+^-TCY) (A), AuNPs (B), and MCH/TBA (C). As seen, there are some sphere-like particles on the p(Cu^2+^-TCY) modified electrode (Figure 1A-a,A-b), and the average roughness of the surface is determined to be 10.4 nm with the largest height of 37.87 nm on the measured region (Figure 1A-c). After AuNPs were anchored, lots of small and uniform particles were observed (Figure 1B-a,B-b). The average surface roughness was decreased to 16.6 nm, while the largest height increased to 59.31 nm (Figure 1B-c). This result confirms that the AuNPs with the smaller and even size have been anchored on the p(Cu^2+^-TCY) modified electrode. After the AuNPs/p(Cu^2+^-TCY) modified electrode was further assembled with the mixing molecule layer of TBA and MCH, some particles were observed on the topographic image (Figure 1C-c), and much more peaks with larger height were found on the three-dimensional image (Figure 1C-b). The average roughness shows a slight increase to 19.8 nm, while the largest height was significantly increased to 76.79 nm (Figure 1C-c). This is rational because the long-chain aptamer molecules have been grafted on the electrode surface.

The successful in-situ growth of Cu^2+^-TCY polymer was further testified by ATR-FTIR, as it is applicable to characterize the interface with the infrared activity. Figure S1 shows the typical ATR-FTIR of the prepared p(Cu^2+^-TCY)/AuE. Clearly, some typical absorption bands are obtained on the electrode. The bands at 1463 and 1574 cm^−1^ are connected to the -C=N of the triazine ring. The weak bands at 746 and 2252 cm^−1^ are ascribed to the -C-S, -C-N bond, and -S-H bonds of TCY, respectively. It is noticeable that the TCY can be easily converted to the thrithioketonal form [27,28], and thus the -C=S group has also appeared at the absorption bands of 1238, 1271, and 1254 cm^−1^. All these characteristic absorption peaks confirm that the TCY-based polymer has been formed on the electrode.

### 3.2. Electrochemical Characterization

**Figure 2:**
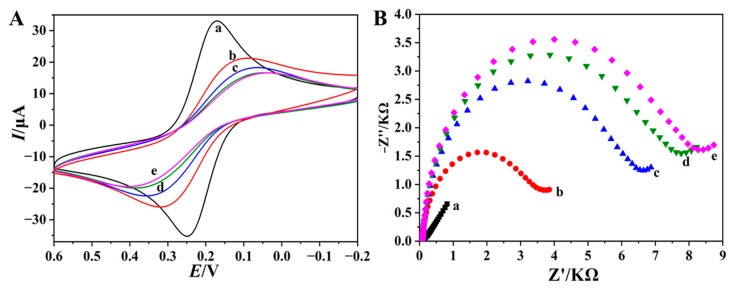
CV (**A**) and EIS (**B**) plots of 0.1 M KCl containing 1.0 mM [Fe(CN)_6_]^3−/4−^ at AuE (a), p(Cu^2+^-TCY)/AuE (b), AuNPs/p(Cu^2+^-TCY)/AuE (c), and MCH/TBA/AuNPs/p(Cu^2+^-TCY)/AuE (d).

The preparation process of the p(Cu^2+^-TCY)-based aptasensing interface was characterized by the electrochemical technologies of CV and EIS using the electroactive molecules of [Fe(CN)_6_]^3−/4−^ as the probe. From the CV results, as displayed in Figure 2A, it is clearly found that [Fe(CN)_6_]^3−/4−^, at the bare AuE, shows a pair of well-defined redox peaks with a small peak-to-peak separation (Δ*E*_p_) of 0.081 V, corresponding to the electron transfer between iron(II)/iron(III) in [Fe(CN)_6_]^3−/4−^ (curve a), suggesting good electronic conductivity of the cleaned AuE. When the [Fe(CN)_6_]^3−/4−^ solution was tested with p(Cu^2+^-TCY)/AuE, the redox peaks were dramatically suppressed, accompanied by the increase in the Δ*E*_p_ (curve b), indicating that the non-conductive p(Cu^2+^-TCY) has been modified on the AuE and inhibited the electron transfer process of [Fe(CN)_6_]^3−/4−^ on the electrode surface. After further assembly of the AuNPs, it is observed that the redox peaks of [Fe(CN)_6_]^3−/4−^ continue to decrease (curve c). This may be due to the existence of negatively charged citrate groups on AuNPs, which leads to electrostatic repulsion between the negatively charged AuNPs and to [Fe(CN)_6_]^3−/4−^ [29]. What is more, when MCH/TBA was anchored, the CV curve shows that the redox peaks of Fe(CN)_6_^3−/4−^ decrease obviously (curve d), which is reasonable that the negatively charged phosphate backbone of TBA causes the electrostatic repulsion to [Fe(CN)_6_]^3−/4−^ [30]. These changes demonstrate that the p(Cu^2+^-TCY)-based aptasensing interface has been successfully fabricated.

Electrochemical impedance spectroscopy (EIS) is a facile and powerful method to probe the change of electrochemical property of electrode interface [31,32]. Therefore, this technology is widely utilized for characterizing the fabrication process of an electrochemical biosensor. The Nyquist plot mode from the EIS measurement commonly has a semicircle part at the high-frequency region and a linear part at the low frequency region [33], and the semicircle diameter at the high-frequency region indicates the electron transfer resistance (*R*_ct_) of the electrochemical reaction at the electrode surface. The typical Nyquist plots of different electrodes are shown in Figure 2B. It is observed that the bare AuE exhibits a invisible semicircle at the high-frequency region (curve a), which is a typical characteristic of electrochemical diffusion process. When p(Cu^2+^-TCY) and AuNPs were assembled on the electrode in turn, the *R*_ct_ values were increased to 3.6 kΩ (curve b) and 6.5 kΩ (curve c), respectively, due to the repulsion and blocking effect of p(Cu^2+^-TCY) and AuNPs toward the [Fe(CN)_6_]^3−/4−^. Furthermore, when PBA and MCH-mixed layer were assembled on the electrode surface, the *R*_ct_ value further increased to 7.8 kΩ (curve d), confirming the successful immobilization of biosensing elements of PBA on the AuNPs/p(Cu^2+^-TCY)/AuE. Therefore, the CV and EIS characterization results are consistent, and both of them indicate the successful fabrication of the aptasensor.

### 3.3. Voltammetric Behavior of Aptasensor and Its Response to Thrombin

**Figure 3:**
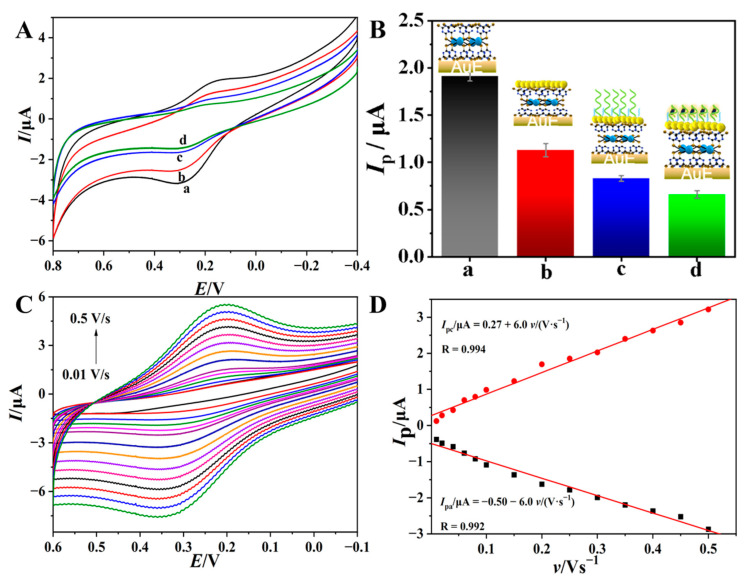
CVs (**A**) of p(Cu^2+^-TCY)/AuE (a) upon successive assembly of AuNPs (b), MCH/TBA (c) and binding with thrombin (d) and the corresponding histogram of oxidation peak currents (*I*_p_) of each electrode (**B**). (**C**) CVs of p(Cu^2+^-TCY)/AuE at different scan rates ranging from 0.01 V s^−1^ to 0.5 V s^−1^ in 0.01 M PBS (pH = 6.80) and (**D**) the corresponding relationships of peak currents (*I*_p_) versus scan rate (*ν*).

The Cu^2+^-based materials usually show intense electrochemical responses due to the fast electron communication and electron transfer kinetics of Cu^2+/^Cu^+^ [34,35,36]. In this work, the electrochemical behavior of the p(Cu^2+^-TCY)-based aptasensor and its voltammetric response for the target molecule of thrombin were investigated by CV. The CV results of each electrode and the corresponding oxidation peak currents (*I*_p_) are shown in Figure 3A,B, respectively. As seen, the p(Cu^2+^-TCY)/AuE shows a pair of obvious redox peaks at 0.18 V and 0.32 V, corresponding to the electron transfer couple of Cu^2+^/Cu^+^ [37,38]. The well-defined redox signal and the moderate redox peak potential are appropriate for the biosensing.

According to the integrated charge (2.1 μC) of the oxidation peak and Faraday’s law [39], the apparent surface concentration of Cu^2+^ (*Г*_Cu_^2+^) adsorbed on the electrode surface was calculated to be 310 pmol cm^−2^, which is about 16-fold larger than the theoretical maximum coverage of monolayer of the electroactive molecule (18.9 pmol cm^−2^) [40], suggesting the presence of multiple layers of electro-active Cu^2+^ center on the electrode surface. This is also a characteristic of an electroactive polymer. The rich electroactive centers are also beneficial to improve the electrochemical signal output as well as the sensitivity of the biosensor. Upon modification of AuNPs, the redox peaks of p(Cu^2+^-TCY) decreased (curve b), which suggested that the negatively charged AuNPs cover on the p(Cu^2+^-TCY) and inhibit the electron transfer process of Cu^2+^/Cu^+^ [41]. After further immobilization of the TBA/MCH layer, the redox peaks continue to decrease (curve c) due to the increase in insulating film thickness on the electrode surface.

The electrochemical sensing feasibility of the aptasensor to thrombin was further investigated by soaking the aptasensor in a thrombin solution and then performing a voltammetric test in blank PBS. The result shows that the redox peaks from the p(Cu^2+^-TCY) show a further decrease (curve c). This change suggests that the aptasensor can recognize the target thrombin molecules to form the nonconductive complex of TBA-thrombin, which change the microenvironment of the electrolyte near the electrode surface and suppress the electrochemical behavior of the p(Cu^2+^-TCY). This result also demonstrates that the in-situ assembled p(Cu^2+^-TCY) on the electrode surface can act as an inherent signal resource of the aptasensor to indicate the bio-affinity reaction.

In order to determine the control factor of electron transfer on p(Cu^2+^-TCY)/AuE, the influence of scan rate (*v*) on the CVs was investigated, and the results are depicted in Figure 3C. As seen, the redox peaks of p(Cu^2+^-TCY)/AuE increase as the *v* values increase from 10 mV s^−1^ to 500 mV s^−1^, and the redox peak currents (*I*_p_) present good linearity with *v* within the scan rate scope (Figure 3D). The above results indicate that the electrochemical behavior of the surface of the modified electrode is controlled by the adsorption process [42,43], confirming the successful modification of electroactive p(Cu^2+^-TCY) on the electrode.

### 3.4. Optimization of Experimental Conditions

The fabrication process of the pp(Cu^2+^-TCY)-based aptasensor and bioaffinity time between aptamer strands and target thrombin were optimized to improve the analytical performance of the biosensor. As shown in Figure S2A, when the assembly cycles of Cu^2+^-TCY increased from 1 to 3, the peak currents (*I*_p_) increased gradually, indicating that increasing amounts of Cu^2+^-TCY molecules have been immobilized on AuE. When the cycle increased from 3 to 5, the peak current values began to decrease, likely due to the fact that the increase in the film thickness limits the electrode communication of p(Cu^2+^-TCY) with the AuE. Therefore, 3 cycles were chosen for the assembly of electroactive Cu^2+^-TCY. Figure S2B shows the relationship between the electrochemical impedance value (*R*_et_) of the biosensor versus the immobilized time (h) of AuNPs. As shown, when immobilization time (*t*_1_) increase from 0 to 4 h, the electrochemical impedance value increase and then level off, indicating the adsorption saturation of AuNPs on the p(Cu^2+^-TCY). Therefore, 4 h was selected as the optimal time for the immobilization of AuNPs. Figure S2C shows the relationship between the *R*_ct_ values of the aptasensor versus the immobilized time of TBA. As shown, the *R*_ct_ values increase when immobilization time (*t*_2_) increases from 0 to 30 min and then level off, indicating that the immobilization reaction between AuNPs and TBA can be finished within 30 min on the electrode surface. Therefore, 30 min was selected as the optimal time for immobilization reaction in the subsequent fabrication assays of the aptasensor. Figure S2D shows the relationship between the oxidation peak currents (*I*_p_) versus the incubation time of the aptasensor in the thrombin solution. Clearly, the values of *I*_p_ decrease when the immobilization time increase from 0 to 40 min and then keep constant, indicating that the reaction between thrombin and TBA reached equilibrium within 40 min. Therefore, 40 min was applied as the optimal reaction time for the capture of thrombin by the aptasensor.

### 3.5. Aptasensing Performance Assessment of the Biosensor

**Figure 4:**
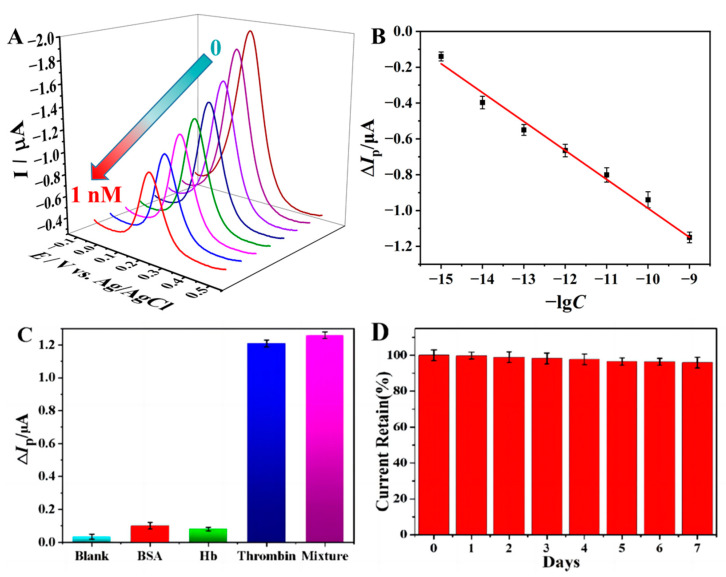
(**A**) DPVs of the aptasensor in PBS buffer after incubation in thrombin solution with concentrations ranging from 0 to 1 nM, and (**B**) the linear relationship between the peak current (*I*_p_) versus the negative logarithm of thrombin concentration (−lg*C*). (**C**) Histogram of the peak current difference (Δ*I*_p_) of the aptasensor after incubation in blank buffer, BSA, Hb, thrombin, and their mixture. The concentration of thrombin is 1.0 fM, and the two control proteins are 10 pM. (**D**) Histogram of the peak current retained after storage of the prepared aptasensor for one week.

Under the optimized conditions, the analytical performance of the developed aptasensor to thrombin was assessed by DPV. Figure 4A shows the DPVs of p(Cu^2+^-TCY)-based aptasensor upon interaction with different concentrations of thrombin. As seen, the oxidation peaks decreased gradually with the increase in the thrombin concentrations, testifying that the electrochemical response of the aptasensor is dependent on the concentration of thrombin. Figure 4B reveals a good linear correlation between the oxidation peak current difference (Δ*I*_p_) and the negative logarithm value of *C* (log*C*) over the range from 1.0 fM to 1.0 nM. The linear regression equation was Δ*I*_p_(μA) = −2.62+0.131log*C* (M), with a correlation coefficient (R) of 0.9937. The detection limit was estimated to be 0.26 fM based on the signal-to-noise ratio (S/N = 3).

Table S1 lists the analytical performance comparison of the prepared aptasensor with previously reported thrombin aptasensors. As seen, the proposed aptasensor exhibits a higher sensitivity and a lower detection limit, showing that the proposed aptasensor is more appropriate for the sensitive analysis of the target thrombin. The excellent analytical performance can be attributed to the following reasons: (1) the high loading density (310 pmol cm^−2^) of the electroactive Cu^2+^ center endows the aptasensor with more intense electroactivity and the electrochemical response, in comparison with the single signal molecule-labeled aptasensors; (2) the use of electrode-confined p(Cu^2+^-TCY) as the inherent signal source and the direct contact of p(Cu^2+^-TCY) with the basic electrode makes the aptasensor having sensitive response to the bioaffinity reaction-induced external microenvironment variation; (3) the immobilization of bioprobe (TBA) on the hydrophilic p(Cu^2+^-TCY) makes the capture of TBA to target thrombin being accomplished in a quasi-homogeneous reaction that has higher efficiency than conventional heterogeneous bioaffinity reaction.

The specificity is a critical factor to assess the performance of the biosensors. In order to explore the specificity of the proposed aptasensor, blank PBS and two kinds of proteins (BSA and Hb) are chosen as controls. The DPVs results and the corresponding histogram of the peak current difference (Δ*I*_p_) of the aptasensor after incubation in blank buffer, BSA, Hb, thrombin, and their mixture are depicted in Figure S3 and Figure 4C, respectively. The results show that both of the two control proteins with the concentrations of 10 pM cannot cause significant signal changes as the same with the blank PBS. However, the presence of 1.0 fM thrombin in the test solution induced a significant change in the voltammetric signal, and even the 10 pM control proteins of BSA and Hb coexist in the thrombin solution (1.0 fM). These results imply that the aptasensor offers high specificity toward thrombin.

The reproducibility of the developed aptasensor was investigated by detecting a 0.1 pM target with five parallel-made electrodes, and the results are shown in Figure S4. The data show that the peak currents’ relative standard deviation (RSD) values of the aptasensors before and after interaction with 0.1 pM thrombin are 7.9% and 6.2%, respectively. When a signal change (Δ*I*_pa_) was applied, an RSD value of 3.6% was estimated for the five parallel-made aptasensors showing that the sensing strategy has an acceptable reproducibility for thrombin detection. The stability of the aptasensor was examined by storage of the prepared aptasensor in a refrigerator (4 °C) and then testing its electrochemical signal every day. The result shows that the electrochemical signal remains at 91.5% after storage for 7 days (Figure 4D), indicating that the aptasensor has good chemical stability when stored in a low-temperature environment. The excellent electrochemical stability can be ascribed to the high chemical stability of the inorganic-organic Cu^2+^-TCY polymer and the tight immobilization of aptamer on AuNPs via the stable Au-S bond.

### 3.6. Determination of Thrombin in Actual Serum Samples

The practical application capacity of the proposed atasensor for thrombin analysis was evaluated by the determination of thrombin spiked into the human serum samples. The results reveal that when 0.05 nM, 0.5 nM, and 1.0 nM thrombin were respectively injected into 2-fold diluted serum samples and measured five times for each sample, the recoveries and RSD were in the range of 97.2–103.0% and 4.2–6.3% (Table S2), respectively. These results indicate that the developed sensing method has good precision and reliability for thrombin analysis in real samples.

## 4. Conclusions

In summary, a novel electrochemical thrombin sensing platform has been designed and constructed based on the use of Cu^2+^-TCY polymer as the inherent sensing signal. The electroactive inorganic-organic polymer of p(Cu^2+^-TCY) was facilely in-situ prepared on the AuE via the repetitive immersing of AuE in TCY and Cu^2+^ solution. Then, the rich -SH group on the p(Cu^2+^-TCY) was utilized as the functional site for the attachment of AuNPs. After further assembly of thiolated thrombin-specific aptamer, a novel aptamer bearing inherent electrochemical activity was achieved. The aptasensor realizes the integration of the electrochemical signal source and the immobilization matrix of the bioprobe. After the binding of the aptamer on the electrode surface with the target, the physical and chemical situation of the interface changed, resulting in the variation of the electrochemical signal of the p(Cu^2+^-TCY), and therefore the target can be table-free detected. The proposed aptasensor provides a new strategy for the construction of sensitive and facile electrochemical aptasensor.

## Data Availability

The data that support the findings of this study are available from the corresponding author upon reasonable request.

## Supplementary Materials

The following supporting information can be downloaded at: https://www.mdpi.com/article/10.3390/bios13050532/s1, Figure S1: The ATR-FTIR image of p(Cu^2+^-TCY); Figure S2: Experimental condition optimization; Figure S3: Specificity of aptasensor; Figure S4: Reproducibility of the aptasensor; Table S1: Comparison of analytical performance of the proposed aptasensor with the reported ones. Table S2: Determination of thrombin in serum samples using the proposed biosensor. References [19,44,45,46,47,48] are citied in the Supplementat Materials.
