# Supplementary material for: In-Situ Fabrication of Electroactive Cu2+-Trithiocyanate Complex and Its Application for Label-Free Electrochemical Aptasensing of Thrombin
Source: Biosensors (Basel). 2023 May 10;13(5):532. doi: 10.3390/bios13050532 (PMC10216145; doi:10.3390/bios13050532)
Supplement: Supplementary file 1 [file biosensors-13-00532-s001.zip › biosensors-2360472-supplementary.pdf]

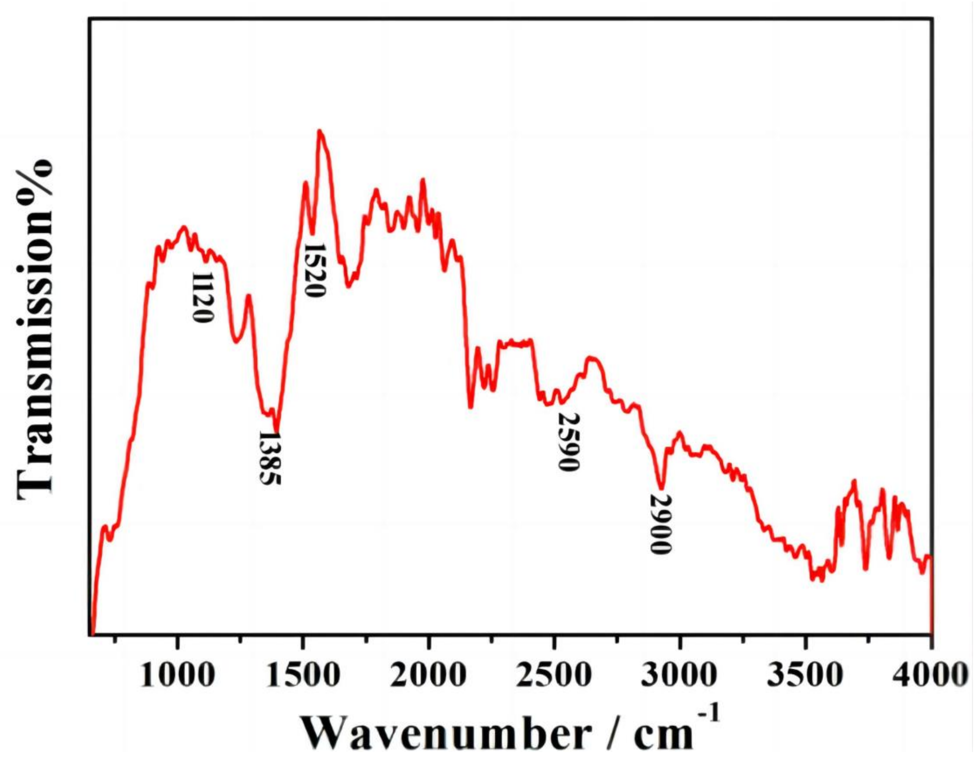

Figure S1. The ATR-FTIR image of p(Cu^{2+}-TCY)

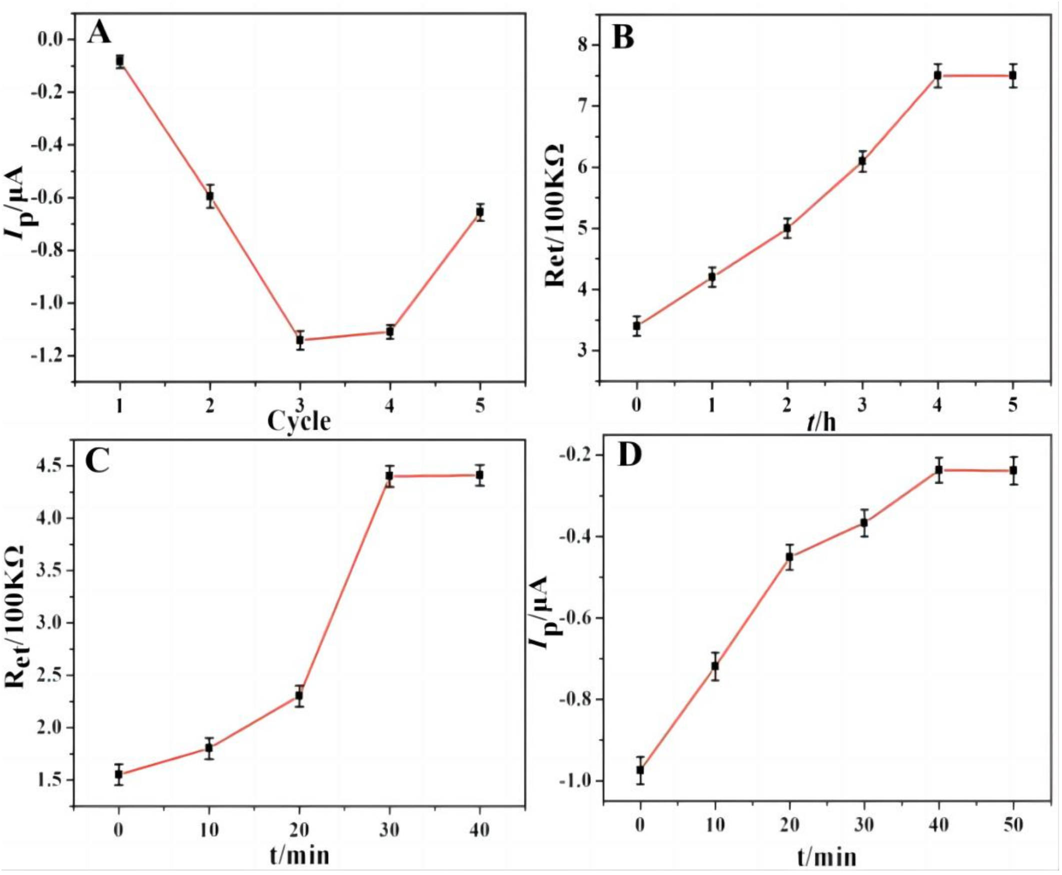

Figure S2. (A) Effect of self-assembly cycles of Cu^{2+} and TCY on the peak currents (I_p), (B) Effect of immobilization time (t) on AuNPs on the R_{ct} values of the electrode, (C) Effect of immobilization time (t) of TBA on the R_{ct} values of the electrode, and (D) Effect of binding time (t) of thrombin on the peak currents (I_p) of the aptasensor

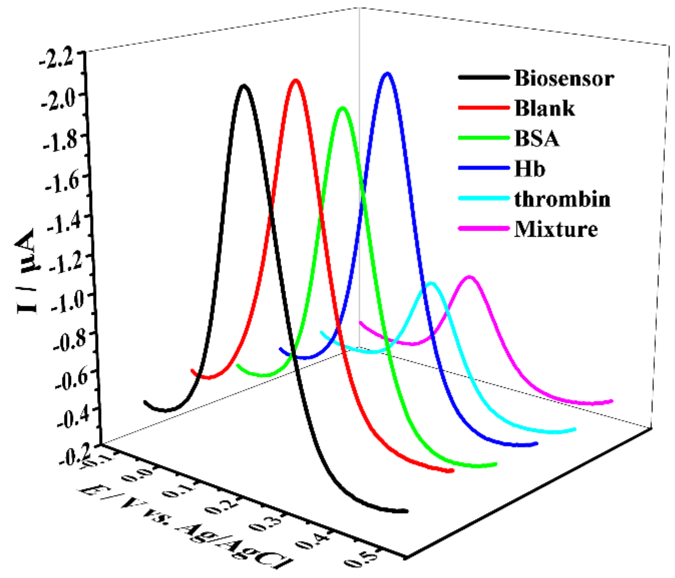

Figure S3. DPVs of the aptasensor after incubation in blank buffer, 10 pM BSA, 10 pM Hb, 1.0 fM thrombin and their mixture.

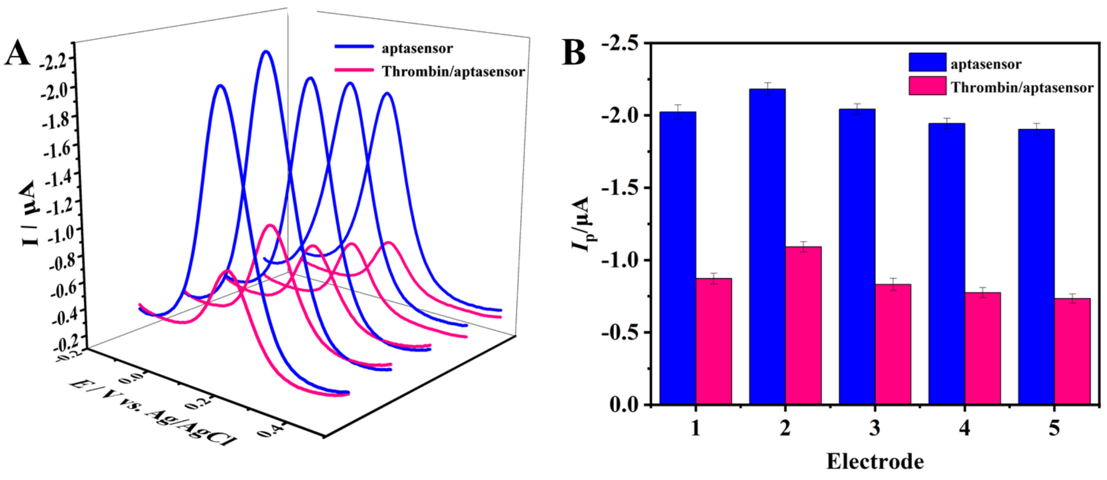

Figure S4. Reproducibility of the aptasensor

Table S1 Comparison of analytical performance of the proposed aptasensor with the previously reported thrombin aptasensors

Materials	Methods	Linear ranges	Limit of Detection (fM)	Refers.
Fe_2O_3/graphene	DPV	10 pM to 4.0 nM	1000	[44]
AuNPs-MXene	DPV	5 fM to 1 pM	1.7	[45]
AgNPs/GO	SWV	50 pM to 5 nM	3.0×10^4	[46]
C_{60}/MWCNTs-PEI	DPV	50 fM to 20 nM	5	[47]
Ag-g-C_3N_4	EIS	100 fM to 20 nM	38	[48]
Au-CeO_2	DPV	0.5 pM to 30 nM	250	[19]
Cu^{2+}-trithiocyanate complex	DPV	1.0 fM to 1.0 nM	0.26	This work

Table S2 Determination of thrombin in serum samples using the proposed biosensor

Serum samples	Added (nM)	Detected (nM)	Recovery
1	0.0500	0.0493	98.6%
2	0.500	0.486	97.2%
3	1.00	1.03	103%
